# A novel drug specific mRNA biomarker predictor for selection of patients responding to dovitinib treatment of advanced renal cell carcinoma and other solid tumors

**DOI:** 10.1371/journal.pone.0290681

**Published:** 2023-08-30

**Authors:** Steen Knudsen, Anker Hansen, Marie Foegh, Steen Petersen, Hana Mekonnen, Lin Jia, Preeti Shah, Victoria Martin, Gregory Frykman, Roberto Pili

**Affiliations:** 1 Allarity Therapeutics, Hørsholm, Denmark; 2 Allarity Therapeutics, Boston, MA, United States of America; 3 Amarex Clinical Research, Germantown, MD, United States of America; 4 Bethesda, MD, United States of America; 5 Jacobs School of Medicine, Buffalo, NY, United States of America; University of Granada: Universidad de Granada, SPAIN

## Abstract

**Purpose:**

Dovitinib is a receptor tyrosine kinase inhibitor of VEGFR1-3, PDGFR, FGFR1/3, c-KIT, FLT3 and topoisomerase 1 and 2. The drug response predictor (DRP) biomarker algorithm or DRP-Dovitinib is being developed as a companion diagnostic to dovitinib and was applied retrospectively.

**Patients and methods:**

Archival tumor samples were obtained from consenting patients in a phase 3 trial comparing dovitinib to sorafenib in renal cell carcinoma patients and the DRP-Dovitinib was applied. The biomarker algorithm combines the expression of 58 messenger RNAs relevant to the *in vitro* sensitivity or resistance to dovitinib, including genes associated with FGFR, PDGF, VEGF, PI3K/Akt/mTOR and topoisomerase pathways as well as ABC drug transport, and provides a likelihood score between 0–100%.

**Results:**

The DRP-Dovitinib divided the dovitinib treated RCC patients into two groups, sensitive (n = 49, DRP score >50%) or resistant (n = 86, DRP score ≤ 50%) to dovitinib. The DRP sensitive population was compared to the unselected sorafenib arm (n = 286). Median progression-free survival (PFS) was 3.8 months in the DRP sensitive dovitinib arm and 3.6 months in the sorafenib arm (hazard ratio 0.71, 95% CI 0.51–1.01). Median overall survival (OS) was 15.0 months in the DRP sensitive dovitinib arm and 11.2 months in the sorafenib arm (hazard ratio 0.69, 95% CI 0.48–0.99). The observed clinical benefit increased with increasing DRP score. At a cutoff of 67% the median OS was 20.6 months and the median PFS was 5.7 months in the dovitinib arm. The results were confirmed in five smaller phase II trials of dovitinib which showed a similar trend.

**Conclusion:**

The DRP-Dovitinib shows promise as a potential biomarker for identifying advanced RCC patients most likely to experience clinical benefit from dovitinib treatment, subject to confirmation in an independent prospective trial of dovitinib in RCC patients.

## Introduction

Targeted therapies against the VEGF and mTOR signaling pathways previously represented the standard and second-line treatment options for patients with metastatic renal cell carcinoma (mRCC) [1]. The acquired resistance remains a significant challenge for both RCC and other solid tumors. Nearly all patients who initially respond to these treatments acquire resistance, and there is an unmet medical need for new agents targeting angiogenesis and tumor growth in patients with renal cell carcinoma that was previously treated with at least two systemic therapies including VEGF targeted therapies and mTOR inhibitors [2]. One of the proposed escape mechanisms from VEGF-targeted therapies is the FGF pathway activation that appears to be a molecular mechanism by which resistance develops in RCC [3]. Patients with renal cell carcinoma who previously received VEGF-targeted therapies, show increased plasma FGF2 concentrations [4]. Additionally, increased FGF2 concentrations are reported as a prognostic factor for metastasis and survival [5]. Therefore, targeting anti-angiogenic escape with FGF pathway inhibition, is one of the potential strategies for patients who progress on anti-VEGF therapies.

Immune checkpoint inhibitors have emerged as an effective treatment against advanced RCC [6]. As opposed to therapies selectively targeting angiogenic pathways, immune checkpoint (PD1-PD-L1/CTLA4) inhibitors work to directly reverse the adaptive camouflage tumor cells deploy to avoid host immunity [7]. As with targeted therapies, there is evidence that primary resistance to immunotherapies may form in some tumor microenvironments and adaptive resistance developing in others [8]. There is an unmet medical need for treatment following first and second line treatment with these established and effective drugs.

Despite these advances, the majority of unselected patients will not respond to many of these treatments, and there are no current predictive biomarkers for renal cell carcinoma patients. There is a need to identify biomarkers that enrich for patients most likely to respond.

The biomarker DRP-Dovitinib is being developed as a companion diagnostic to select patients with advanced renal cell carcinoma and other solid tumors for therapy with the multi-TKI, dovitinib. This predictive biomarker is an assay, that based on samples from a tumor estimates the likelihood of that tumor responding to a specific drug. The DRP (Drug Response Predictor) method builds on the comparison of sensitive and resistant cell lines including transcriptomic information from the National Cancer Institute (NCI) NCI60 cell lines and clinical tumor biology in a systems biology network. Messenger RNA (mRNA) is used to make a drug specific estimate. The validity of the DRP platform has been demonstrated in a number of published studies covering retrospective analysis of published data, blinded prospective analysis of archival samples and prospectively conducted trials [9–15].

The primary objective of this study was to validate the DRP-Dovitinib biomarker and its ability to select patients that benefit from treatment with dovitinib.

## Materials and methods

### Patients

In the GOLD multicenter, open-label, randomized phase 3 trial (clinicatrials.gov identifier NCT01223027; [16]), eligible patients were randomly assigned through an interactive voice and web response system to receive dovitinib (500 mg orally according to a 5-days-on and 2-days-off schedule) or sorafenib (400 mg orally twice daily) in a 1:1 ratio. Randomization was stratified by risk group and region. Patients had metastatic renal cell carcinoma with clear cell or a component of clear cell histology and had received at least one previous VEGF-targeted therapy and at least one previous mTOR inhibitor. In addition, previous treatment with anticancer therapies, including cytokines and anticancer vaccines, was permitted and washout periods varied from 2 weeks to 6 weeks depending on the agent. The patients (aged ≥18 years) must have had disease progression on or within 6 months of last therapy. Additional inclusion criteria included measurable disease (Response Evaluation Criteria in Solid Tumors [RECIST], version 1.1), Karnofsky performance status of 70 or greater, and adequate hematological, renal, and hepatic functions. Exclusion criteria included previous sorafenib or dovitinib treatment, brain metastases, clinically significant cardiac diseases, or uncontrolled hypertension. Archival tumor blocks/slides were required. Archival tumor samples were collected at screening. If paraffin blocks from multiple biopsies taken from different times were available the most recent biopsy was collected. The trial was conducted between 2011 and 2014 in 27 countries.

In order to test whether the DRP-Dovitinib was independent of tumor type it was evaluated in five non RCC phase II trials.

Hepatocellular carcinoma patients in the Asia-Pacific region were randomized to frontline treatment with dovitinib (N = 82) or sorafenib (N = 83) in a phase II study (NCT01232296, [17]). Pre-treatment biopsies were available from 8 consenting patients in the dovitinib arm and analyzed.

Metastatic endometrial cancer patients with progressive disease after first-line chemotherapy were treated with dovitinib (N = 53) in a phase II study (NCT01379534, [18]). Pre-treatment biopsies were obtained from 35 consenting patients and analyzed.

The multicenter phase II trial DOVIGIST evaluated dovitinib as a second-line treatment of patients (N = 39) with gastrointestinal stromal tumor (GIST) refractory to imatinib or who do not tolerate imatinib (NCT01478373, [19]). Pre-treatment biopsies were obtained from 25 consenting patients, and 19 passed lab QC criteria (RNA yield) for analysis.

HER-2 negative metastatic breast cancer patients (N = 81) were treated with dovitinib in a phase II trial (NCT00958971, [20]). Patients had a median of 2 prior chemotherapy regiments and a median of 2 prior hormone therapy regimens. Pre-treatment biopsies were obtained from 57 consenting patients, and 44 passed lab QC criteria (RNA yield) for analysis.

Postmenopausal patients (N = 97) with hormone receptor positive HER-2 negative, advanced breast cancer that had progressed during or after prior endocrine therapy were randomized to receive fulvestrant plus dovitinib (N = 47) or placebo (N = 50) (NCT01528345, [21]). Pre-treatment biopsies were obtained from 26 consenting patients in the dovitinib arm, and 21 passed lab QC criteria (RNA yield) for analysis.

### Ethics

The protocols and informed consent, including consent to publish and optional consent to future research with stored samples, have been approved by Institutional Review Board/Independent Ethics Committee/Research Ethics Board in all countries where the studies were conducted. S1 Table lists the clinical trial sites and the name of the ethics committee that provided approval for each site.

The studies were conducted according to the ethical principles of the Declaration of Helsinki and in compliance with Good Clinical Practice, including the archiving of essential documents.

Informed consent was obtained from each patient in writing before study entry.

The studies used coded and not individually identifiable leftover specimens previously collected from individuals who have given informed consent to have their biopsies analyzed.

### DRP development

In order to develop a DRP specific to the drug dovitinib, the drug was submitted to the NCI for testing in the NCI60 panel. The sensitivity of the 60 cell lines (including 8 renal cancer cell lines) measured as growth inhibition GI_50_ ranged from 42 nM to 10 mM. This observed difference in sensitivity was correlated to the observed baseline gene expression in the 60 cell lines and 58 genes were identified as positively correlated (Pearson CC>0.25) or negatively correlated (Pearson CC<-0.25).

The predicted sensitivity to dovitinib is based on the difference between the mean of the 30 positively correlated probesets and the mean of the 28 negatively correlated probesets measured in formalin fixed paraffin embedded (FFPE) tumor tissue with the Affymetrix array HG-U133_Plus2 and normalized with the R based Robust Multiarray Average. The resulting score is compared to a reference population of 92 primary and metastatic clear cell renal cell carcinoma patients and expressed as a percentile of the reference population. The percentile is the DRP score. Based on experience with other DRPs, the cutoff was chosen as the median of the reference population, i.e. a score of 50%. The device was locked before application to the clinical samples in this study. An explorative analysis of efficacy was performed with a higher cutoff of 67%.

For tumor types other than RCC, the same 58 genes were used, but the cutoff was redefined as the median of a reference population of the same tumor type. For breast cancer, the reference population was 819 diagnostic breast cancer biopsies. For tumor types such as hepatocellular carcinoma (HCC), endometrial cancer and gastrointestinal stromal tumor (GIST) one reference population was used that consisted of all three tumor types.

### Statistical analysis

As this is a retrospective analysis, there is a risk that choices are influenced by knowledge of the clinical outcome. To avoid this, the device was applied to clinical samples according to Instructions for Use without access to information about the clinical outcome of each patient. Clinical outcome was combined with the DRP results by an independent statistician. No formal statistical sample size calculation was performed. A Statistical Analysis Plan was finalized before the independent biostatistician received the data. The primary analysis was the difference in PFS according to a logrank test comparing DRP positive patients in the dovitinib arm to unselected sorafenib arm (i.e. all patients in the sorafenib arm) in the efficacy evaluable population. Secondary analysis included OS and ORR. Hazard ratios with continuous DRP scores were calculated on a unit scale using coxph(Surv ~ score) in R. Meta-analysis of all cohorts was performed with the metagen function in the R package meta, where individual studies were pooled via a random effect inverse-variance meta-analysis model that combines the log hazard ratios weighted by their standard error.

### Analytical validation

The DRP-Dovitinib assay was analytically validated to ensure that it is fit for clinical use. Among the parameters quantified were input amount, sample stability, sample type, tumor cell content, necrosis, reproducibility, sensitivity and specificity, and linearity.

## Results

### Pathways probed by the DRP biomarker

**Fig 1:**
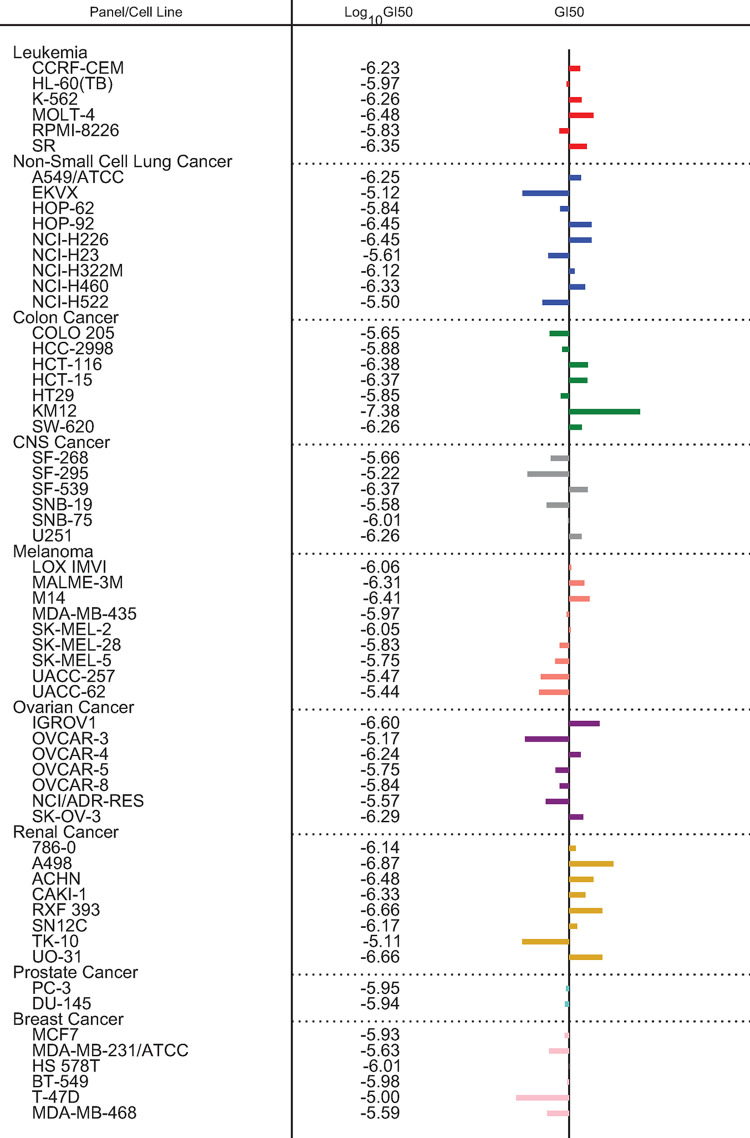
Measured growth inhibition of cancer cell lines by dovitinib.

The compound dovitinib was tested in the NCI60 panel of cell lines. Measured growth inhibition is shown in Fig 1. Thirty mRNAs had a baseline expression pattern that was relevant to the *in vitro* sensitivity (correlation between expression and sensitivity > 0.25) and 28 mRNAs were relevant to resistance (correlation < -0.25) of dovitinib. The biomarker algorithm combines the expression of these 58 genes in a DRP-Dovitinib likelihood score between 0–100%. The mRNAs that are identified by the Dovitinib DRP have in the literature been associated with a number of pathways relevant to the action of dovitinib, and some mRNAs have been associated with more than one pathway. This includes genes associated with FGFR, PDGF, VEGF, PI3K/Akt/mTOR and topoisomerase pathways as well as ABC drug transport (Table 1). Genes in black are those overexpressed in dovitinib sensitive cell lines (positive correlation), genes in *italic* are those that are overexpressed in dovitinib resistant cell lines (negative correlation). However, about half of the genes have no published link to the known biology of dovitinib action in tumor cells (grouped as Other).

**Table 1:** Genes of the DRP-Dovitinib grouped by pathway.

Pathway/process	Genes
**FGF**	SEL1L3 LSM4 STAT6—*GATA3*
**VEGF**	VEGFA FABP5 DPP4 LDLR TMSB10 P4HA1 STAT6 RPL38—*HRAS UCP2 ATM GATA3 SPDEF CUL3*
**PDGF**	STAT6 UCP2*—TUG1 ATM GATA3*
**PI3K/Akt/mTOR**	REDD1 TRIM22 FABP5 MCT4 INSIG1—*HRAS BAG5 SCAMP3 IGFBP5 ATM SRM TOB1 CUL3 CERS2*
**Drug resistance**	*ABCF1 IGFBP5*
**Metastasis and hypoxia**	DPP4 REDD1—*ATM NDUFV1 CKB*
**Topoisomerase**	*HRAS APITD1*
**Other**	ZNF395 RPS20 TPK1 MIR196B HSPE1 CAV2 APOL1 PTPRE DPYSL2 ANP32B RPL27A EPB41L2 RPL3—*BAG6 MARCH6 EMC3 CLPTM1 MAGEA1 LDOC1 ZNF331 MCUR1 NBPF10 PPP1R11*

PI3K/Akt/mTOR is downstream of FLT3, c-KIT and PDGFRA.

The 58 probesets in the DRP correspond to 54 unique genes, because 4 probesets probe different regions of genes already probed by other probesets.

### Evaluation of DRP biomarker in the phase III renal cell carcinoma trial

**Fig 2:**
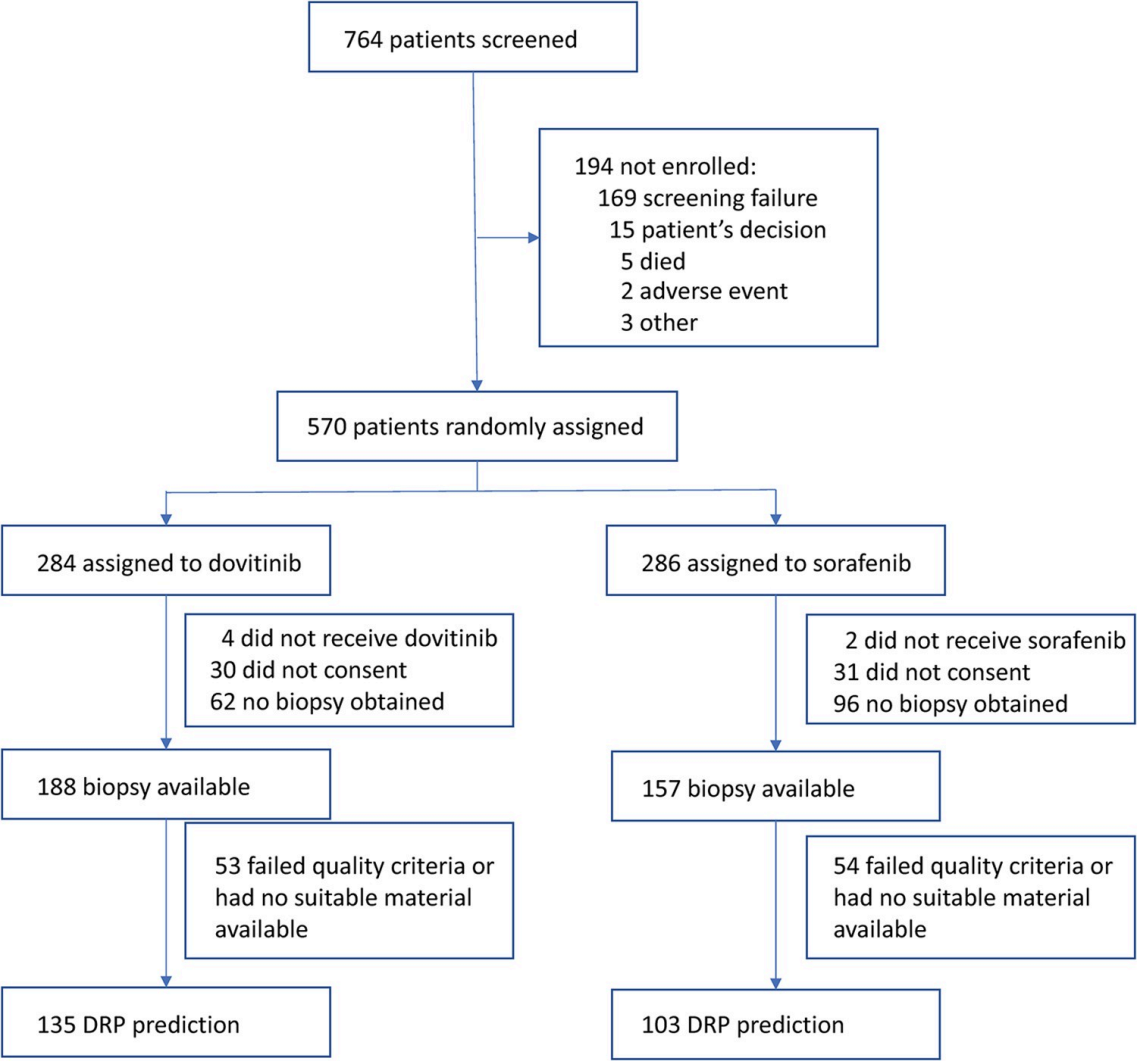
Disposition of patients in the GOLD phase III trial.

The phase III GOLD trial of dovitinib versus sorafenib in renal cell carcinoma [16] was used for validation of the DRP-Dovitinib. The DRP-Dovitinib algorithm was locked before blinded validation, ensuring that no clinical data were used in its development. Patient disposition is shown in Fig 2, including biopsy availability. Baseline characteristics for the dovitinib and sorafenib arms and the biopsy available subsets are shown in S1–S4 Tables.

The DRP-Dovitinib-RCC divided the dovitinib treated patients in two groups, sensitive (n = 49, DRP score >50%) or resistant (n = 86, DRP score ≤ 50%) to dovitinib. The DRP sensitive population was compared to the unselected sorafenib arm (n = 286) in Table 2. Median progression-free survival (PFS) was 3.8 months in the DRP sensitive dovitinib arm and 3.6 months in the sorafenib arm (hazard ratio 0.71, 95% CI 0.51–1.01). Median overall survival (OS) was 15.0 months in the DRP sensitive dovitinib arm and 11.2 months in the sorafenib arm (hazard ratio 0.69, 95% CI 0.48–0.99). Unconfirmed overall response rate (ORR) was 14.3% in the DRP sensitive dovitinib arm (95% CI 6.41–27.9) and 7.7% in the sorafenib arm (95% CI 5.0–11.6).

**Table 2:** Efficacy parameters in the selected dovitinib arm compared to the unselected sorafenib arm.

Efficacy Parameter	Dovitinib	Sorafinib	p-value	HR
	DRP Score >50%	Unselected		(95% CI)
	N = 49	N = 286		
Median PFS, Months	3.75	3.61	0.057	0.71
(95% CI)	(3.68, 5.39)	(3.48, 3.71)		(0.51,1.01)
Median OS, Months	15.0	11.2	0.04	0.69
(95% CI)	(12.94, 26.25)	(9.66, 13.37)		(0.47,0.99)
Unconfirmed ORR	14.3%	7.7%	0.14	-
(95% CI)	(6.41, 27.9%)	(5.0, 11.6%)		

The DRP sensitive population was also compared to the DRP resistant population. Median progression-free survival (PFS) was 3.8 months in the DRP sensitive population and 3.8 months in the DRP resistant group (hazard ratio 0.73, 95% CI 0.49–1.09). Median overall survival (OS) was 15.0 months in the DRP sensitive group and 9.1 months in the DRP resistant group (hazard ratio 0.60, 95% CI 0.39–0.91). Unconfirmed overall response rate (ORR) was 14.3% in the DRP sensitive group (95% CI 6.41–27.9) and 9.3% in the DRP resistant group (95% CI 4.39–18.0). Table 3 shows efficacy parameters PFS, OS and ORR above and below cutoff in the dovitinib arm.

**Table 3:** Efficacy parameters above and below cutoff in the dovitinib arm.

Efficacy Parameter	Dovitinib	Dovitinib	p-value	HR
	DRP Score >50%	DRP Score ≤50%		(95% CI)
	N = 49	N = 86		
Median PFS, Months	3.75	3.75	0.12	0.73
(95% CI)	(3.68, 5.39)	(2.14, 3.91)		(0.49,1.09)
Median OS, Months	15.0	9.13	0.02	0.60
(95% CI)	(12.94, 26.25)	(7.49, 13.2)		(0.39,0.91)
Unconfirmed ORR	14.3%	9.3%	0.38	-
(95% CI)	(6.41, 27.9%)	(4.39, 18.0%)		

Dichotomizing the prediction above and below a cutoff is associated with a loss in statistical power. If continuous DRP scores are used to calculate PFS hazard ratios it is improved to 0.54 (95% CI 0.34–0.89) and with OS it is improved to 0.52 (95% CI 0.32–0.85). Pearson correlation between DRP scores and tumor response (encoded as 1 = CR, 2 = PR, 3 = SD, 3 = non-PR/non-PD, 4 = PD) is -0.21 with a one-sided p-value of 0.01. With continuous scores all three efficacy measures become statistically significant.

**Fig 3:**
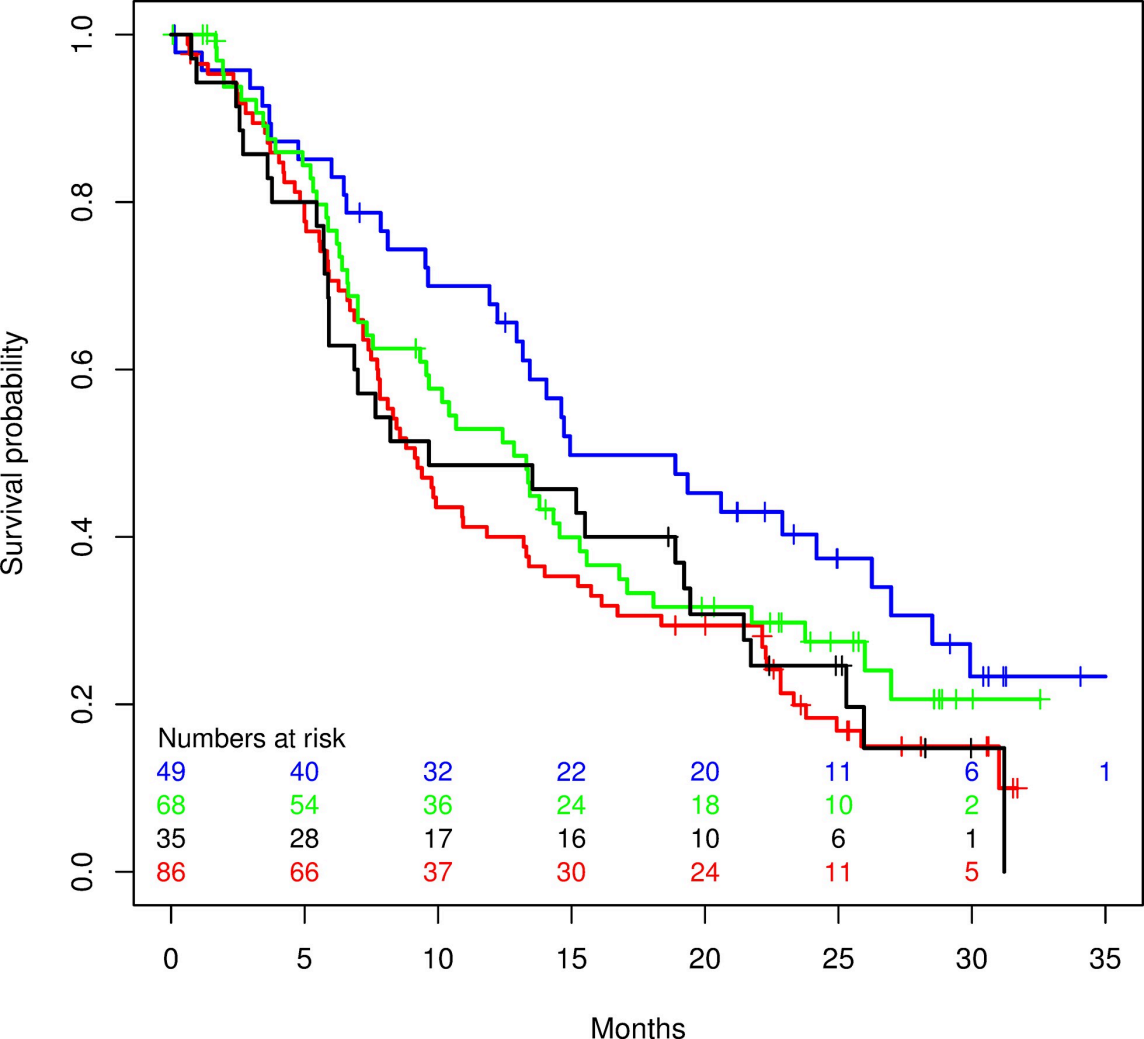
Kaplan-Meier curves of overall survival. DRP-Dovitinib positive (above cutoff, blue) and DRP-Dovitinib negative (red) subsets of the dovitinib treatment arm; DRP-Dovitinib positive (black) and DRP-Dovitinib negative (green) subsets of the sorafenib treatment arm.

DRP-Dovitinib-RCC was also applied to the sorafenib arm. Fig 3 shows that DRP-Dovitinib-RCC fails to identify patients that benefit from sorafenib. Median PFS was 3.6 months in the DRP score >50% sorafenib arm (N = 35) and 3.6 months in the DRP score ≤ 50%) sorafenib arm (N = 68, hazard ratio 0.88, 95% CI 0.56–1.4). Median OS was 9.7 months in the DRP score >50% sorafenib arm and 12.8 months in the DRP score ≤ 50% sorafenib arm (hazard ratio 1.16, 95% CI 0.73–1.9). These data support that the DRP-Dovitinib-RCC is drug specific and is not a prognostic biomarker.

The primary analysis assessed the clinical outcome for patients above the pre-selected cutoff. A more detailed analysis reveals that the clinical benefit from dovitinib treatment is proportional to the DRP score: the higher the DRP score cutoff, the greater the clinical benefit. For patients with a score above 50, the median overall survival is 15.0 months (95% CI 12.9–26.3) while for patients with a score above 60 it has increased to 20.6 months (95% CI 9.5–29.9) and for patients with a score above 70 it has increased to 35.6 months (95% CI 7.9–35.6). Table 4 shows that a choice of a higher cutoff improved all efficacy parameters. The DRP sensitive population (n = 15 of 135) was compared to the entire sorafenib arm (n = 286). Median PFS was 5.7 months in the DRP sensitive population and 3.6 months in sorafenib arm (hazard ratio 0.42, 95% CI 0.21–0.86). Median OS was 20.6 months in the DRP sensitive group and 11.2 months in the sorafenib arm (hazard ratio 0.55, 95% CI 0.28–1.08).

**Table 4:** Efficacy parameters at a cutoff of 67%.

Efficacy parameter	DRP positive dovitinib arm (cutoff = 67%, N = 15)	Entire sorafenib arm (N = 286)	P-value	Hazard ratio
Unconfirmed ORR	20.0%	7.7%	0.11	
(95% CI)	(5.3, 49%)	(5.0, 12%)		
Median PFS	5.7 mo	3.6 mo	0.02	0.42
(95% CI)	(1.9, 20.3 mo)	(3.5, 3.7 mo)		(0.21, 0.86)
Median OS	20.6 mo	11.2 mo	0.08	0.55
(95% CI)	(9.5, 35.6 mo)	(9.7, 13.4 mo)		(0.28, 1.08)

### Effect of necrosis

A pre-planned analysis showed that the DRP was not able to predict outcome based on 56 out of 135 samples that contain more than minimal necrosis. Microdissecting 36 FFPE blocks with more than minimal necrosis after mounting on slides to extract only tumor tissue identified by a pathologist, changed the DRP score and clinical prediction. Table 5 shows a vast improvement in prediction of outcome in the dovitinib arm above and below cutoff after microdissecting tumor tissue, suggesting that if microdissection had been used on all samples with necrosis, the DRP performance shown in Tables 1–4 and Fig 3 would have improved.

**Table 5:** Effect of microdissecting 35 samples with necrosis from the dovitinib arm.

Efficacy parameter	Before microdissection	After microdissection
Unconfirmed ORR above cutoff	8.3%	16.7%
(95% CI)	(0.4, 40.2%)	(0.9, 63.5%)
PFS hazard ratio	1.18	0.42
(95% CI)	(0.51, 2.7)	(0.13, 1.4)
OS hazard ratio	0.96	0.10
(95% CI)	(0.42, 2.21)	(0.01, 0.76)

### MSKCC risk stratification

There already exists a stratification of metastatic RCC patients based on MSKCC risk level. The MSKCC index is based on performance status, LDH, calcium and hemoglobin levels, and time from diagnosis to systemic treatment. The three strata poor, intermediate and favorable risk, were not entirely balanced across the groups compared in this study (S5 Table) but the DRP improved overall survival in all risk strata: poor, intermediate as well as favorable risk strata (S6 Table). The DRP was an independent predictor of survival if risk strata were included as a covariate (HR = 0.50, 95% CI 0.31–0.78 using microdissected samples)

### Safety

Safety data for subjects who belonged to DRP-Dovitinib positive group was compared to the sorafenib unselected group. Incidence of treatment-emerging adverse events (TEAEs), serious adverse events (SAEs), deaths, and other safety parameters were similar between the groups (S7 Table).

### Evaluation of DRP biomarker in five phase II trials

The DRP-Dovitinib was developed based on cell lines from different tumor types and not specific to RCC. To test whether the resulting biomarker was predictive in other solid tumor types as well, biopsies from phase II trials of dovitinib in other tumor types were tested.

**Fig 4:**
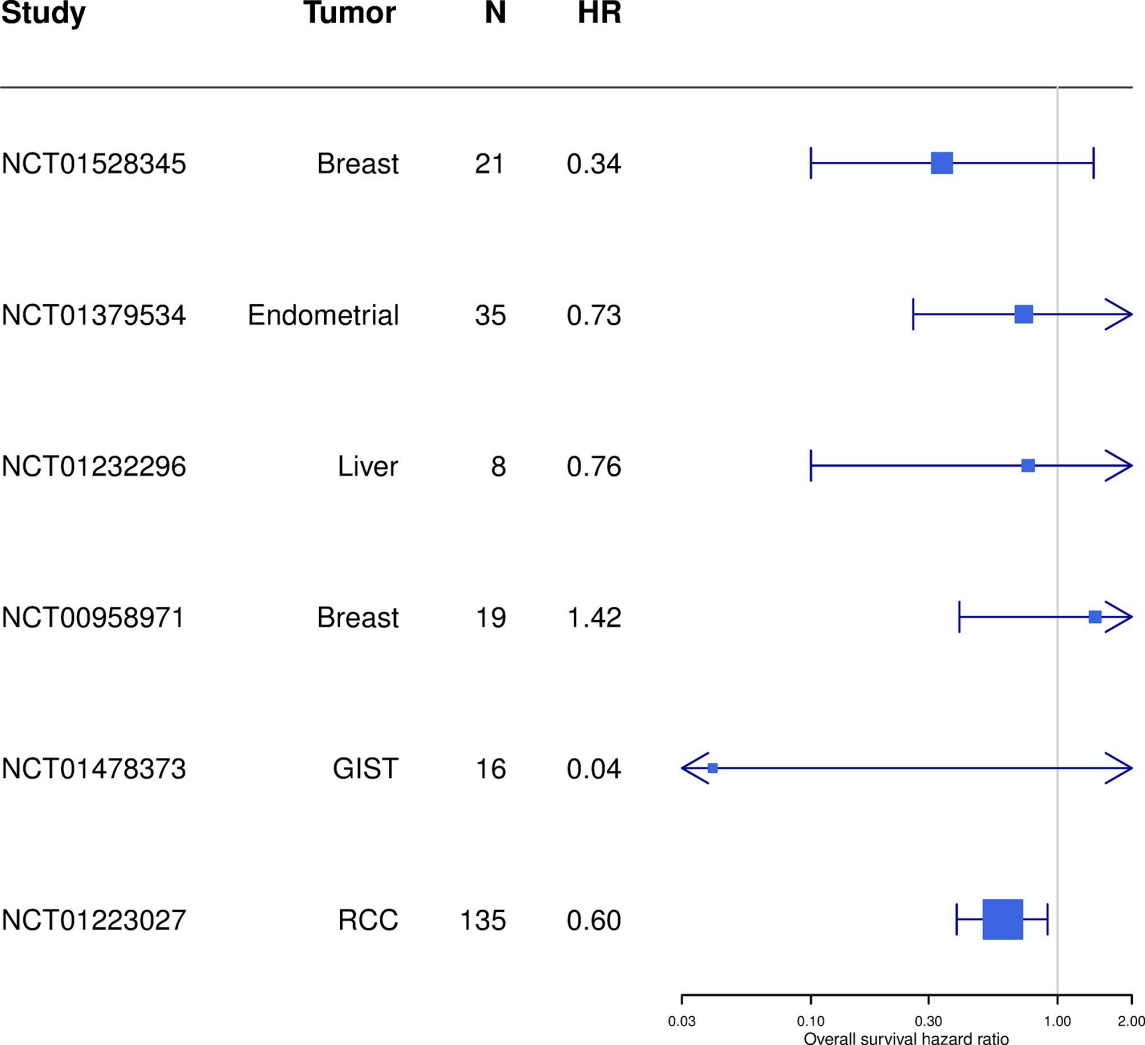
Forest plot of overall survival hazard ratios (HR) of DRP-Dovitinib applied to six cohorts. A hazard ratio of 1.00 means there is no difference in survival between DRP positive and DRP negative patients. The box indicates the hazard ratio and the box size is proportional to its precision. The 95% confidence interval is indicated with vertical lines, while arrows indicate clipping of the confidence interval to the range of the x-axis. A confidence interval that does not include a hazard ratio of 1.0 is statistically significant.

**Fig 5:**
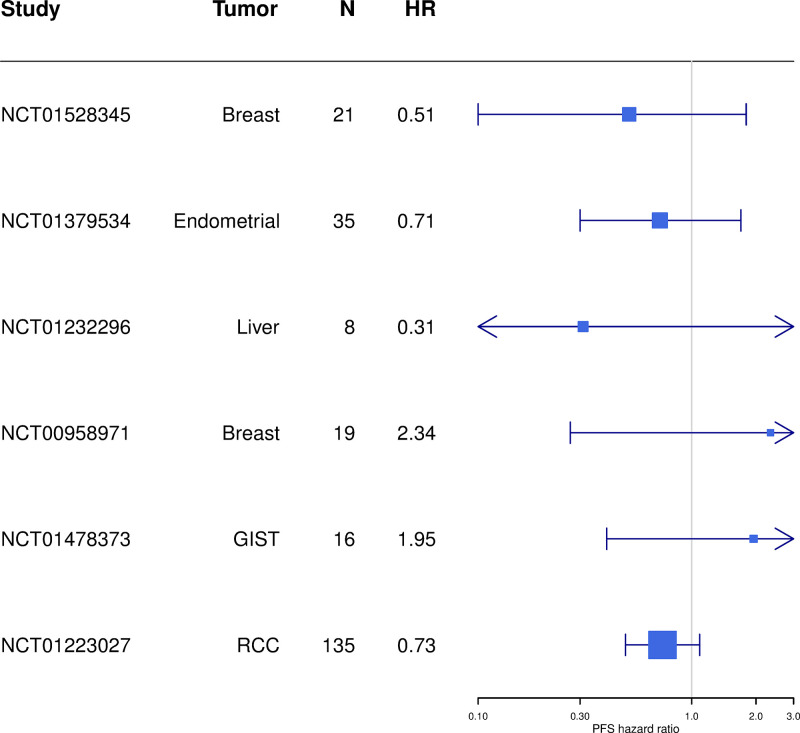
Forest plot of PFS hazard ratios (HR) of DRP-Dovitinib applied to six cohorts. A hazard ratio of 1.00 means there is no difference in survival between DRP positive and DRP negative patients. The box indicates the hazard ratio and the box size is proportional to its precision. The 95% confidence interval is indicated with vertical lines, while arrows indicate clipping of the confidence interval to the range of the x-axis. A confidence interval that does not include a hazard ratio of 1.0 is statistically significant.

The gene list and software used was the same as that used for the RCC trial, but the reference population used to convert the DRP score into a population percentile and median cutoff was specific to each cancer type. Fig 4 shows a Forest plot of the overall survival hazard ratios between DRP positive and DRP negative observed for the six cohorts, including RCC. Fig 5 shows a Forest plot of the progression free survival hazard ratios between DRP positive and DRP negative observed for the six cohorts, including RCC.

## Discussion

Over the years the treatment of RCC has changed dramatically with the introduction of immune check point inhibitors. RTKIs continue to have a major therapeutic role in this disease. More recently, the RTKI tivozanib has been approved for RCC patients treated with 3 lines of therapies and beyond. Dovitinib has been tested in comparison with sorafenib in third line setting of RCC. Overall, the unconfirmed response rate for dovitinib was only around 8% and did not show superior clinical benefit to sorafenib.

To date, the search for a predictive biomarker for RTKIs has been disappointing. Biomarkers based on a single gene or pathway may not be optimal for a drug that targets a large number of kinases such as dovitinib. In endometrial cancer dovitinib treatment effect seemed independent of FGFR2 mutation status (Koneckny 2015). In hormone receptor (HR) positive metastatic breast cancer dovitinib response was associated with FGF pathway amplification (Andre 2013), but this observation was not confirmed in a larger study where FGF pathway amplification was associated with a worse response to dovitinib but a longer PFS (Musolino 2017). In this report we describe a multi-gene biomarker that measures a number of pathways and processes targeted by dovitinib.

The DRP-Dovitinib consists of 58 genes which is more than most other predictive biomarkers that focus on a single target. Looking beyond the target FGFR, the 58 genes also probe the VEGF and PDGF pathways, as well as two potential novel dovitinib drug resistance genes. A number of the 58 genes have yet to be linked to known biology of interaction between dovitinib and tumor cells.

Diagnostic biopsies or archived tumor resection specimens, most from the primary tumor, were used to make predictions in all trials. Thus the tumors have been subject to selection during a number of treatments prior to treatment with dovitinib. Only three biopsies in the RCC dovitinib arm of the GOLD trial were taken after last anticancer treatment, an insufficient number to evaluate the effect of the timing of the tumor sample on prediction of response.

Overall survival differs significantly between patients above the DRP score of 50% and below or equal to 50% cutoff, and the other efficacy parameters show a trend in the same direction. For PFS, the hazard ratio is 0.73, while the median is the same 3.75 months in the two arms. That is because of the fixed tumor scanning interval of 8 weeks. Both arms cross the median during the second scan at 3.75 months. However, before and after the second scan, the proportion of progression-free differ between the two arms.

The most sensitive analysis of DRP-Dovitinib clinical performance is correlation between the quantitative DRP score and a quantitative measure of treatment outcome. Even a semi-quantitative measure such as RECIST categories is correlated to the DRP score in the dovitinib arm. Dichotomization allows estimation of survival above and below cutoff, the measure of most clinical importance. Due to a relatively small sample size compared to the overall population the predictions based on dichotomized scores are only borderline significant in the phase III RCC trial, and not significant in the individual small phase II trials.

The cutoff score of 50% and 67% were selected prospectively, in order to avoid fitting the cutoff to the existing data. The higher the score cutoff, the greater the benefit from treatment with dovitinib. Thus both the binary prediction of whether a biopsy is above cutoff and the actual DRP score are potentially valuable information for the treating physicians and their patients.

The optimal cutoff is a balance between withholding treatment from responding and non-responding patients. This balance was assessed after unblinding in a receiver operating characteristic that confirmed that the pre-specified cutoff of 50% is close to the optimal balance and that no overall improvement can be obtained with a different cutoff.

Treatment with the multi-targeted kinase inhibitor dovitinib along with the novel companion diagnostic DRP-Dovitinib is associated with greater clinical benefit as compared to sorafenib in third line setting for renal cell carcinoma.

Dovitinib and sorafenib are both multi-tyrosine kinase inhibitors, but the kinases they target and their IC_50_ differ [22, 23]. The DRP-Dovitinib biomarker is highly drug specific and does not select responders to sorafenib. Despite DRP-Dovitinib selecting the dovitinib treated patients with longer overall survival it is not prognostic, since it does not select the sorafenib treated patients with longer survival, and it selects improved survival for Dovitinib treated patients in all MSKCC risk strata. Also, if the DRP has merely predicted MSKCC risk, it would not have been an independent predictor in a multivariate survival analysis that include risk strata.

The DRP-Dovitinib signature is not only applicable to renal cell carcinoma. Comparable trends have been obtained in four other tumor types, albeit in much smaller trials. The cutoff between tumors predicted sensitive and tumors predicted resistant must be adjusted between tumor types to account for systemic differences in gene expression.

Overall, our report suggests that the DRP-Dovitinib score may be a predictive tool for the efficacy of dovitinib in patients with mRCC and other solid tumors. The primary endpoint of PFS was not met, but the secondary endpoint OS was statistically significant improved. The relevance of the DRP-Dovitinib score remains to be further determined in patients with RCC.

## Conclusion

Our results suggest that the proposed DRP-Dovitinib biomarker algorithm may identify a subpopulation of RCC patients with increased clinical benefit with dovitinib treatment, supported by similar trends in the majority of five cohorts of other tumor types treated with dovitinib. This is a first example of transcriptomic-based predictive signature in RCC patients randomized to two different RTKIs. These results should be confirmed in an independent prospective trial of dovitinib in RCC patients.

## Supporting information

S1 Table Participating sites and the ethics committee (EC) that approved the trial. (PDF)

S2 Table Baseline demographics. (PDF)

S3 Table Baseline performance status. (PDF)

S4 Table Metastatic sites. (PDF)

S5 Table Balance between MSKCC risk groups in different arms of the study. (PDF)

S6 Table Stratification of median OS by MSKCC risk factor and treatment arm with 95% CI. (PDF)

S7 Table Overview of adverse events. (PDF)

S1 File Anonymous prediction and response data. A comma-separated text file. "-" means not done. Best overall unconfirmed response is encoded as follows: 1 = Complete response, 2 = Partial response, 3 = Stable disease, 4 = Progressive disease, 5 = Non-CR/Non-PD, 997 = Unknown.
(CSV)
