# Supplementary material for: A novel drug specific mRNA biomarker predictor for selection of patients responding to dovitinib treatment of advanced renal cell carcinoma and other solid tumors
Source: PLoS One. 2023 Aug 30;18(8):e0290681. doi: 10.1371/journal.pone.0290681 (PMC10468037; doi:10.1371/journal.pone.0290681)
Supplement: S1 Table — (PDF) [file pone.0290681.s001.pdf]

S1 Table: Participating sites and the Ethics Committee (EC) that approved the trial

Center Name	Center City	Center State/Province	Center Postal Code	Center Country	EC/IRB Name
**Sanatorio de la Providencia**	Buenos Aires		C1050AAK	Argentina	FEFyM - FUNDACION DE ESTUDIOS FARMACOLOGICOS Y DE MEDICAMENTOS PROF. LUIS M. ZIEHER
**Centro Oncologico de Rosario**	Rosario	Sante Fe	S200DSK	Argentina	COMITE DE ETICA DEL CENTRO ONCOLOGICO DE ROSARIO (CECOR)
**The Queen Elizabeth Hospital**	Woodville	SA	5011	Australia	"Ethics of Human Research Committee The Queen Elizabeth Hospital"
**Austin Health, Department of Medical Oncology**	Heidelberg	VIC	3084	Australia	"Austin Health Human Research Ethics Committee Austin Hospital"
**Haem & Oncology Clinics of Australasia, Mater Medical Centre**	South Brisbane	QLD	4101	Australia	Bellberry Human Research Ethics Committee
**Royal North Shore Hospital**	St. Leonards	NSW	2065	Australia	Cancer Institute NSW
**Westmead Hospital**	Westmead	NSW	2145	Australia	Cancer Institute NSW
**Western Hospital**	Footscray	Victoria	3011	Australia	"Ethics of Human Research Committee The Queen Elizabeth Hospital"
**Univ.Klinik für Innere Medzin I**	Wien		1090	Austria	Ethikkommission der Med, Universitat Wien Borschkegasse 8b/E 06
**Allgem. oeffentl. Krankenhaus der Stadt Linz**	Linz		A-4020	Austria	Ethikkommission der Med, Universitat Wien Borschkegasse 8b/E 06
**Gasthuisberg University Hospital**	Leuven		3000	Belgium	"UZ Leuven Herestraat 49"
**Centre Hospitalière Universitaire Sart Tilman**	Liège		4000	Belgium	"CHU Sart-Tilman Batiment B35 Start-Tilman"
**Cliniques Universitaires Saint-Luc**	Bruxelles		1200	Belgium	"Cliniques Universitaires St. Luc Avenue Hippocrate 10"
**Universitair Ziekenhuis Gent**	Gent		9000	Belgium	"UZ Gent DePintelaan 185"
**Hospital São Lucas da PUC-RS**	Porto Alegre	RS	90610-000	Brazil	Comite de Etica em pesquisa da Pontificia Universidade Catolica do rio Grande do Sul
**Sunnybrook Health Sciences Center**	Toronto	ON	M4N 3M5	Canada	Ontario Cancer Research Ethics Board
**St. Joseph's Hospital**	Hamilton	ON	L8N 4A6	Canada	Ontario Cancer Research Ethics Board
**B.C. Cancer Agency**	Vancouver	BC	V5Z 4E6	Canada	UBC BCCA Research Ethics Board
**Cross Cancer Institute**	Edmonton	AB	T6G 1Z2	Canada	Alberta Cancer Research Ethics comittee
**The Ottawa Hospital**	Ottawa	ON	K1H 8L6	Canada	Ontario Cancer Research Ethics Board
**Tom Baker Cancer Centre**	Calgary	Alberta	T2N 4N2	Canada	Alberta Health Services Research Ethics Office
**Princess Margaret Hospital (name changed to Princess Margaret Cancer Centre)**	Toronto	ON	M5G 2M9	Canada	Ontario Cancer Research Ethics Board
**London Regional Cancer Centre**	London	ON	N6A 4L6	Canada	Ontario Cancer Research Ethics Board
**QE II Health Sciences Centre**	Halifax	NS	B3H 2Y9	Canada	Capital Health Research Ethics Board
**CHUM - Hopital Notre-Dame**	Montreal	QC	H2L 4M1	Canada	Comité d'éthique de la recherche CHUM

**Les Urologues Associés du CHUM**	Montreal	QC	H2X 1N8	Canada	Institutional Review Board Services
**Jewish General Hospital**	Montreal	QC	H3T 1E3	Canada	McGill Faculty of Medicine Institutional Review Board
**Saskatoon Cancer Centre**	Saskatoon	SK	S7N 4H4	Canada	University of Saskatchewan
**Fundación Cardioinfantil**	Bogotá			Colombia	Comite de Etica en invesdtigacion Clinica de la Fundaction Cardioinfantil-Instituto de Cardiologia
**Masarykuv onkologicky institut**	Brno		656 53	Czech Republic	Etická komise Masarykova onkologického ústavu
**Fakultni Nemocnice Olomouc**	Olomouc		775 20	Czech Republic	Etická komise Fakultní nemocnice Olomouc a LF UP Olomouc (MEC)
**Fakultní nemocnice V Motole**	Praha		150 06	Czech Republic	Etická komise pro multicentrické klinické hodnocení FN v Motole
**Institut Gustave Roussy**	Villejuif Cedex		94805	France	CPP Ile de France VII Hopital de Bicetre
**Hôpital Foch**	Suresnes		92150	France	CPP Ile de France VII Hopital de Bicetre
**Hopital Albert Michallon**	Grenoble		38043	France	CPP Ile de France VII Hopital de Bicetre
**Centre Francois Baclesse**	Caen Cedex		14021	France	CPP Ile de France VII Hopital de Bicetre
**Centre René Gauducheau**	Saint-Herblain Cédex		44805	France	CPP Ile de France VII Hopital de Bicetre
**Centre Eugène Marquis**	Rennes Cedex		35062	France	CPP Ile de France VII Hopital de Bicetre
**Hopital Minjoz**	Besancon Cedex		25030	France	CPP Ile de France VII Hopital de Bicetre
**Hopital de Hautepierre**	Strasbourg Cedex		F-67098	France	CPP Ile de France VII Hopital de Bicetre
**Hôpital Saint André**	Bordeaux Cedex		33075	France	CPP Ile de France VII Hopital de Bicetre
**Institut Claudius Regaud**	Toulouse Cedex 3		31052	France	CPP Ile de France VII Hopital de Bicetre
**Institut Paoli Calmettes**	Marseille		13273	France	CPP Ile de France VII Hopital de Bicetre
**Centre Léon Bérard**	Lyon Cedex		69373	France	CPP Ile de France VII Hopital de Bicetre
**Centre Alexis Vautrin**	Vandoeuvre-Les-Nancy Cedex		54511	France	CPP Ile de France VII Hopital de Bicetre
**Institut de Cancérologie de la Loire**	Saint Priest en Jarez Cedex		42271	France	CPP Ile de France VII Hopital de Bicetre
**Hôpital Européen Georges Pompidou**	Paris		75015	France	CPP Ile de France VII Hopital de Bicetre
**Hôpital Pitie Salpetriere**	Paris		75651	France	CPP Ile de France VII Hopital de Bicetre
**Centre Antoine Lacassagne**	Nice Cedex 2		06189	France	CPP Ile de France VII Hopital de Bicetre
**Kliniken der Med. Hochschule Hannover**	Hannover		30625	Germany	Ethik-Kommission der Medizinischen Hochschule Hannover
**Klinikum Nuernberg Nord**	Nuernberg		90419	Germany	"Friedrich-Alexander-Universität Erlangen-Nürnberg Medizinische Fakultät Ethik-Kommission"
**Humboldt-Univ. Charité Campus Mitte**	Berlin		10117	Germany	"Landesamt für Gesundheit und Soziales Ethik-Kommission des Landes Berlin"
**Gemeinschaftspraxis Dr. Klausmann/Dr. Welslau**	Aschaffenburg		63739	Germany	Ethik-Kommission der Bayerischen Landesärztekammer
**Phillips Universitat Marburg**	Marburg		35033	Germany	Phillips Universitat Marburg Fachbereich Medizin Dekanat Ethikkomission Baldingstrasse
**Universitaetsklinikum Hamburg-Eppendorf**	Hamburg		20246	Germany	"Ärztekammer Hamburg Ethik-Kommission"
**Univ.-Klinikum Heidelberg**	Heidelberg		69120	Germany	Ethik-Kommission der Medizinischen Fakultät Heidelberg

**Univ-Klinikum Leipzig**	Leipzig		04103	Germany	Ethik-Kommission an der Medizinischen Fakultät der Universität Leipzig
**Praxis Dr. Kube**	Chemnitz		09119	Germany	"Sächsische Landesärztekammer Ethik-Kommission"
**Universitatsklinkum Ulm**	Ulm		89075	Germany	"Universität Ulm Ethik-Kommission"
**Univ.-Klinikum Greifswald**	Greifswald		17475	Germany	"Universitätsmedizin Greifswald Ethikkommission Institut für Pharmakologie"
**Univ.-Klinikum Münster**	Muenster		48149	Germany	Ethikkommission der Ärztekammer Westfalen-Lippe und der Medizinischen Fakultät der Westfälischen Wilhelms-Universität Münster
**Klinikum Weiden**	Weiden		92637	Germany	Ethik-Kommission der Bayerischen Landesärztekammer
**Friedrich-Schiller-Universität Jena**	Jena		07743	Germany	"Universitätsklinikum Jena Ethik-Kommission"
**Universitätsklinikum Erlangen-Nürnberg**	Erlangen		91054	Germany	"Friedrich-Alexander-Universität Erlangen-Nürnberg Medizinische Fakultät Ethik-Kommission"
**General Hospital of Athens ALEXANDRA**	Athens		11528	Greece	National Ethics Committee
**SOTIRIA General Hospital**	Athens		11527	Greece	National Ethics Committee
**Euromedica General Clinic of Thessaloniki**	Thessaloniki		54645	Greece	National Ethics Committee
**Orszagos Onkologiai Intezet**	Budapest		H-1122	Hungary	Egeszsegugyi Tudomanyos Tanacs Klinikai Farmakologiai Etikai Bizottsag
**Semmelweis University, Budapest Urology Clinic**	Budapest		1086	Hungary	Egeszsegugyi Tudomanyos Tanacs Klinikai Farmakologiai Etikai Bizottsag
**JNSZ Megyei Hetenyi Geza Korhaz-Rendelointezet**	Szolnok		H-5000	Hungary	Egeszsegugyi Tudomanyos Tanacs Klinikai Farmakologiai Etikai Bizottsag
**Pécsi Tudományegyetem, Onkotherápiás Intézet**	PECS		7624	Hungary	Egeszsegugyi Tudomanyos Tanacs Klinikai Farmakologiai Etikai Bizottsag
**Debreceni Egyetem, Orvos es Egeszsegtudomanyi Centrum**	Debrecen		4032	Hungary	Egeszsegugyi Tudomanyos Tanacs Klinikai Farmakologiai Etikai Bizottsag
**The Chaim Sheba Medical Center**	Ramat Gan		52621	Israel	The Chaim Sheba Medical Center Ethics commitee
**Assaf Harofeh Medical Center**	Zrifin		70300	Israel	Assaf Harofeh Medical Center Ethics commitee
**Rabin Medical Center**	Petach Tikva		49100	Israel	Rabin Medical Center Ethics commitee
**Azienda Ospedaliera S. Camillo-Forlanini**	Roma	RM	00152	Italy	Comitato Etico Dell'Azienda Ospedaliera San Camillo-C. Forlanini Di Roma
**Azienda Ospedaliera di Rilievo Nazionale A. Cardarelli**	Napoli	NA	80131	Italy	Comitato Etico Dell'Azienda Ospedaliera A. Cardarelli Napoli
**A.O.Univ.Policl.di Modena Univ.Studi Modena e R.Emilia**	Modena	MO	41100	Italy	Comitato Etico Della Provincia Di Modena
**Fodazione IRCCS Policlinico S.Matteo Univ.degli Studi Pavia**	Pavia	PV	27100	Italy	Comitato Di Bioetica Della Fondazione IRCCS Policlinico San Matteo Di Pavia

**Istituti Ospitalieri di Cremona**	Cremona	CR	26100	Italy	Comitato Etico Dell'Azienda Ospedaliera Istituti Ospitalieri Di Cremona
**IRCC - Fondazione del Piemonte per l'Oncologia Univ.Torino**	Candiolo	TO	10060	Italy	Comitato Etico Dell'Azienda Ospedaliera Universitaria S. Luigi Gonzaga Di Orbassano
**Ist. Scien.Romagnolo per lo studio e la cura dei Tumori-IRST**	Meldola	FC	47014	Italy	Comitato Etico Di Area Vasta Romagna E Istituto Scientifico Romagnolo Per Lo Studio E La Cura Dei Tumori Di Meldola
**Presidio Ospedaliero Area Aretina Nord S. Donato**	Arezzo	AR	52100	Italy	Comitato Etico Per Sperimentazione Dei Medicinali Della Ausl
**Fondazione IRCCS - Istituto Nazionale dei Tumori**	Milano	MI	20133	Italy	Comitato Etico Indipendente Della Fondazione IRCCS Istituto Nazionalke Dei Tumori Di Milano
**Keio University Hospital**	Shinjuku-ku	Tokyo	160-8582	Japan	Institutional review board of Keio University Hospital
**Kinki University Hospital**	OsakaSayama	Osaka	589-8511	Japan	Institutional review board of Kinki University Hospital
**Kobe University Hospital**	Kobe	Hyogo	650-0017	Japan	Institutional review board of Kobe University Hospital of medical supplies and medical equipment
**Cancer Institute Hospital of JFCR**	Koto	Tokyo	135-8550	Japan	Institutional review board of Cancer Institute Hospital of JFCR
**Kochi Medical School Hospital**	Nangoku	Kochi	783-8505	Japan	Institutional review board of Yamagata University Hospital
**University Hospital, Kyoto Prefectural Univ. of Medicine**	Kyoto	Kyoto	602-0841	Japan	Institutional review board of University Hospital, Kyoto Prefectural Univ. of Medicine
**Hiroshima University Hospital**	Hiroshima	Hiroshi ma	734-8551	Japan	Institutional review board of Hiroshima University Hospital
**Osaka University Hospital**	Suita-city	Osaka	565-0871	Japan	Institutional review board of Osaka University Hospital
**Nagoya University Hospital**	Nagoya	Aichi	466-8560	Japan	Institutional review board of Nagoya University Hospital
**Chiba Cancer Center**	Chiba	Chiba	260-8717	Japan	Institutional review board of Chiba Cancer Center
**Kyushu University Hospital**	Fukuoka-city	Fukuok a	812-8582	Japan	Institutional review board of of Kyushu University Hospital
**Osaka Medical Center for Cancer and Cardiovascular Diseases**	Osaka	Osaka	537-8511	Japan	Institutional review board of Osaka Medical Center for Cancer and Cardiovascular Diseases
**Tokyo Women's Medical University Hospital**	Shinjuku-ku	Tokyo	162-8666	Japan	Institutional review board of Tokyo Women's Medical University Hospital
**Osaka City University Hospital**	Osaka-city	Osaka	545-8586	Japan	Institutional review board of Osaka City University Hospital
**Saitama Medical University International Medical Center**	Hidaka	Saitama	350-1241	Japan	Institutional review board of Saitama Medical University International Medical Center
**Yokohama Minami Kyousai Hospital**	Yokohama-city	Kanaga wa	236 0037	Japan	Institutional review board of Yokohama Minami Kyousai Hospital
**Kobe City Medical Center General Hospital**	Kobe	Hyogo	650-0047	Japan	Institutional review board of Kobe City Medical Center General Hospital
**Saitama Cancer Center Hospital**	Kitaadachi-gun	Saitama	338-8553	Japan	Institutional review board of Saitama Cancer Center Hospital
**Kanagawa Cancer Center**	Yokohama	Kanaga wa	241-0815	Japan	Institutional review board of Kanagawa Cancer Center
**Hokkaido P.W.F.A.C Obihiro-Kosei General Hospital**	Obihiro	Hokkaid o	080-0016	Japan	Institutional review board of Hokkaido P.W.F.A.C Obihiro-Kosei General Hospital

**Osaka Medical College Hospital**	Takatsuki	Osaka	569-8686	Japan	Institutional review board of Osaka Medical College Hospital
**Ehime University Hospital**	Toon	Ehime	791-0295	Japan	Institutional review board of Ehime University Hospital
**Shinshu University Hospital**	Matsumoto	Nagano	390-8621	Japan	Institutional review board of Shinshu University Hospital
**Nippon Medical School Hospital**	Bunkyo-ku	Tokyo	113-8603	Japan	Institutional review board of Nippon Medical School Hospital
**Toranomon Hospital**	Minato-ku	Tokyo	105-8470	Japan	Toranomon Hospital and Toranomon Hospital Kajigaya Institutional Review Board
**Severance Hospital**	Seoul		120-752	Korea	Yonsei University Health System
**Asan Medical Center**	Seoul		738-736	Korea	Asan Medical Center
**Seoul National University Hospital**	Seoul		110 744	Korea	Seoul National University Hospital
**Samsung Medical Center**	Seoul		135-710	Korea	Samsung Medical Center
**Erasmus Medisch Centrum (Daniel Den Hoed Kliniek)**	Rotterdam		3075 EA	Netherlands	Erasmus MC
**VU Medisch Centrum**	Amsterdam		1081 HV	Netherlands	Erasmus MC
**Academisch Ziekenhuis Maastricht**	Maastricht		6229 HX	Netherlands	Erasmus MC
**Albert Schweitzer Ziekenhuis**	Dordrecht		3318AT	Netherlands	Erasmus MC
**Leiden University Medical Center**	Leiden		2300 RC	Netherlands	Erasmus MC
**Amphia Ziekenhuis**	Breda		4818 CK	Netherlands	Erasmus MC
**Kreftavdelingen, Helse-Sunnmore HF, Ålesund sjukehus**	Ålesund		NO-6026	Norway	REK Sør-Øst D
**Haukeland Universitetssykehus**	Bergen		NO-5021	Norway	REK Sør-Øst D
**Wojskowy Instytut Medyczny**	Warszawa		04-141	Poland	Komisja Bioetyczna-WIM
**Klinika Nowotworów Uk¿adu Moczowego Centrum Onkologii Instyt**	Warszawa		02-781	Poland	Komisja Bioetyczna-WIM
**King Faisal Specialist Hospital & Research Center Riyadh**	Riyadh		11211	Saudi Arabia	Research Ethics Committee -King Faisal Specialist Hospital & Research Center, Takhssuy St
**Narodny Onkologicky Ustav**	Bratislava	Slovak Republic	83310	Slovakia	Eticka Komisia Narodny onkologicky ustav (NOU)
**Hospital Vall D'Hebron**	Barcelona	Cataluña	08035	Spain	Unitat de Suport al ECIC JUVH (SCEI), Vall d'Zhebron Institut de Recerca (VHIR)
**Hospital Clinic I Provincial De Barcelona**	Barcelona	Cataluña	08036	Spain	Unitat de Suport al ECIC JUVH (SCEI), Vall d'Zhebron Institut de Recerca (VHIR)
**Hospital Durans I Reynals-ICO**	Hospitalet de LLobregat	Cataluña	08907	Spain	Unitat de Suport al ECIC JUVH (SCEI), Vall d'Zhebron Institut de Recerca (VHIR)
**Hospital del Mar**	Barcelona	Cataluña	08003	Spain	Unitat de Suport al ECIC JUVH (SCEI), Vall d'Zhebron Institut de Recerca (VHIR)
**Hospital de la Santa Creu i Sant Pau**	Barcelona	Cataluña	08041	Spain	Unitat de Suport al ECIC JUVH (SCEI), Vall d'Zhebron Institut de Recerca (VHIR)

**HOSPITAL UNIVERSITARIO GERMANS TRIAS I PUJOL**	Badalona	Cataluña	08916	Spain	Unitat de Suport al ECIC JUVH (SCEI), Vall d'Zhebron Institut de Recerca (VHIR)
**Instituto Valenciano de Oncologia (I.V.O)**	Valencia	Comunidad Valenciana	46009	Spain	Unitat de Suport al ECIC JUVH (SCEI), Vall d'Zhebron Institut de Recerca (VHIR)
**HOSPITAL FUNDACION ALCORCON**	Alcorcón	Madrid	28922	Spain	Unitat de Suport al ECIC JUVH (SCEI), Vall d'Zhebron Institut de Recerca (VHIR)
**CONSORCI HOSPITALARI PARC TAULI**	Sabadell	Cataluña	08208	Spain	Unitat de Suport al ECIC JUVH (SCEI), Vall d'Zhebron Institut de Recerca (VHIR)
**Hospital 12 De Octubre**	Madrid		28041	Spain	Unitat de Suport al ECIC JUVH (SCEI), Vall d'Zhebron Institut de Recerca (VHIR)
**Hospital Gregorio Marañón**	Madrid		28007	Spain	Unitat de Suport al ECIC JUVH (SCEI), Vall d'Zhebron Institut de Recerca (VHIR)
**Hospital Clinico San Carlos**	Madrid	Madrid	28040	Spain	Unitat de Suport al ECIC JUVH (SCEI), Vall d'Zhebron Institut de Recerca (VHIR)
**Hospital Nuestra Senora De Valme**	Dos Hermanas	Andalucía	41700	Spain	Unitat de Suport al ECIC JUVH (SCEI), Vall d'Zhebron Institut de Recerca (VHIR)
**Hospital Virgen de la Victoria**	Málaga	Andalucía	29010	Spain	Unitat de Suport al ECIC JUVH (SCEI), Vall d'Zhebron Institut de Recerca (VHIR)
**Hospital general de Asturias**	Oviedo	Asturias	33006	Spain	Unitat de Suport al ECIC JUVH (SCEI), Vall d'Zhebron Institut de Recerca (VHIR)
**HOSPITAL CLINICO UNIVERSITARIO SANTIAGO DE COMPOSTELA**	Santiago de Compostela	A Coruña	15706	Spain	Unitat de Suport al ECIC JUVH (SCEI), Vall d'Zhebron Institut de Recerca (VHIR)
**Hospital Reina Sofia**	Córdoba	Andalucía	14004	Spain	Unitat de Suport al ECIC JUVH (SCEI), Vall d'Zhebron Institut de Recerca (VHIR)
**H. Universitario Insular de Gran Canaria**	Las Palmas de Gran Canarias	Las Palmas de G.C	35016	Spain	Unitat de Suport al ECIC JUVH (SCEI), Vall d'Zhebron Institut de Recerca (VHIR)
**Complejo Hospitalario de Navarra**	Pamplona	Navarra	31008	Spain	Unitat de Suport al ECIC JUVH (SCEI), Vall d'Zhebron Institut de Recerca (VHIR)
**H. Clínica Benidorm**	Benidorm	Alicante	03501	Spain	Unitat de Suport al ECIC JUVH (SCEI), Vall d'Zhebron Institut de Recerca (VHIR)
**Karolinska Universitetssjukhuset Solna**	Stockholm		SE-171 76	Sweden	Regional Ethics Committée
**Norrlands Universitetssjukhus**	Umeå		SE-901 85	Sweden	Regional Ethics Committée
**Kantonsspital St. Gallen**	St. Gallen		9007	Switzerland	Ethikkommission des Kantons St. Gallen
**Siriraj Hospital**	Bangkok		10700	Thailand	Siriraj Institutional Review Board
**Mount Vernon Hospital**	Northwood	Middlesex	HA6 2RN	United Kingdom	Health Research Authority National Research Ethics Service
**The Christie Hospital**	Manchester	Middlesex	M20 9BX	United Kingdom	Health Research Authority National Research Ethics Service
**Southampton General Hospital**	Southampton		SO16 6YD	United Kingdom	Health Research Authority National Research Ethics Service

**Bristol Haematology and Oncology Centre**	Bristol	Avon	BS2 8ED	United Kingdom	Health Research Authority National Research Ethics Service
**The Leicester Royal Infirmary**	Leicester		LE1 5WW	United Kingdom	Health Research Authority National Research Ethics Service
**St George's Hospital**	London		SW17 0QT	United Kingdom	Health Research Authority National Research Ethics Service
**Royal Free Hospital, Renal Cancer Clinical Trials Unit**	London		NW3 4QG	United Kingdom	Health Research Authority National Research Ethics Service
**Utah Cancer Specialists**	Salt Lake City	UT	84103	US	Quorum Review, Inc.
**University of Minnesota Medical Center - Fairview**	Minneapolis	MN	55455	US	Research Subjects Protection Program University of Minnesota
**UCLA/ University of California Los Angeles**	Los Angeles	CA	90095	US	Office for Protection of Research Subjects
**Cedars Sinai Medical Center**	Los Angeles	CA	90048	US	Cedars-Sinai Medical Center
**Memorial Sloan Kettering Cancer Center**	New York	NY	10065	US	Memorial Sloan-Kettering Cancer Center
**University of Virginia Health Systems**	Charlottesville	VA	22908-0334	US	University of Virginia Institutional Review Board for Health Sciences Research
**University of Kansas Cancer Center**	Westwood	KS	66202	US	Human Subjects Committee University of Kansas Medical Center
**Vanderbilt University Medical Center**	Nashville	TN	37212-3505	US	Vanderbilt University Institutional Review Board
**University of Texas Southwestern Medical Center**	Dallas	TX	75390	US	UT Southwestern
**Highlands Oncology Group**	Fayetteville	AR	72703	US	Quorum Review IRB
**The West Clinic**	Memphis	TN	38120	US	Western Institutional Review Board
**Medical University of South Carolina**	Charleston	SC	29425	US	Medical University of South Carolina,
**St. Luke's Hospital and Health Network**	Bethlehem	PA	18015	US	St Luke's Hospital & Health Network Institutional Review Board
**University of California San Diego**	La Jolla	CA	92093-0698	US	University of California, San Diego, Human Subjects Program
**Florida Cancer Specialists**	Fort Myers	FL	33916	US	Western Institutional Review Board
**Willamette Valley Clinical Studies**	Springfield	OR	97477	US	US Oncology, Inc., Institutional Review Board
**Rocky Mountain Cancer Centers**	Denver	CO	80218	US	US Oncology, Inc., Institutional Review Board
**New York Oncology Hematology, P.C.**	Albany	NY	12206	US	US Oncology, Inc., Institutional Review Board
**Baylor Health Care System / Sammons Cancer Center**	Dallas	TX	75246	US	US Oncology, Inc., Institutional Review Board
**Deke Slayton Cancer Center**	Webster	TX	77598	US	US Oncology, Inc., Institutional Review Board
**Comprehensive Cancer Centers of Nevada**	Las Vegas	NV	89109	US	US Oncology, Inc., Institutional Review Board
**Wayne State University/Karmanos Cancer Institute**	Detroit	MI	48201	US	Wayne State University, Institutional Review Board
**Cooper Hospital, Division of Hematology/Oncologyu**	Voorhees	NJ	08043	US	Cooper University Hospital
**Straub Clinic & Hospital**	Honolulu	HI	96813	US	Western Institutional Review Board
**Kaiser Permanente Hawaii**	Honolulu	HI	96817	US	Kaiser Permanente Hawaii

**Northeast Georgia Cancer Center**	Athens	GA	30607	US	Western Institutional Review Board
**Rockwood Clinic**	Spokane	WA	99216	US	US Oncology, Inc., Institutional Review Board
**Texas Oncology, PA**	Houston	TX	77024	US	US Oncology, Inc., Institutional Review Board
