# Supplementary material for: A novel drug specific mRNA biomarker predictor for selection of patients responding to dovitinib treatment of advanced renal cell carcinoma and other solid tumors
Source: PLoS One. 2023 Aug 30;18(8):e0290681. doi: 10.1371/journal.pone.0290681 (PMC10468037; doi:10.1371/journal.pone.0290681)
Supplement: S2 Table — (PDF) [file pone.0290681.s002.pdf]

S2 Table: Baseline demographics

			Dovitinib			Sorafenib		
Demographics		Units	Assayable N=135	Non-Assayable N=149	All N=284	Assayable N=103	Non-Assayable N=183	All N=286
Age	Mean (SD)	Years	59.7 (11.5)	61.3 (9.2)	60.6 (10.4)	61 (10.2)	61.1 (10.1)	61.1 (10.1)
	Median		61.0	61.0	61.0	62.0	62.0	62.0
	<65		86 (63.7)	101 (67.8)	187 (65.8)	58 (56.3)	107 (58.5)	165 (57.7)
	>=65		49 (36.3)	48 (32.2)	97 (34.2)	45 (43.7)	76 (41.5)	121 (42.3)
Sex	Female	n (%)	32 (23.7)	39 (26.2)	71 (25.0)	24 (23.3)	43 (23.5)	67 (23.4)
	Male		103 (76.3)	110 (73.8)	213 (75.0)	79 (76.7)	140 (76.5)	219 (76.6)
Race	Caucasian		117 (86.7)	116 (77.9)	233 (82.0)	87 (84.5)	145 (79.2)	232 (81.1)
	Black		3 (2.2)	0	3 (1.1)	2 (1.9)	3 1.6)	5 (1.7)
	Asian		15 (11.1)	27 (18.1)	42 (14.8)	14 (13.6)	26 (14.2)	40 (14.0)
	Unknown/ Other		0	6 (4.1)	6 (2.2)	0	9 (4.9)	9 (3.1)
Ethnicity	Hispanic or Latino		4 (3.0)	8 (5.4)	12 (4.2)	2 (1.9)	14 (7.7)	16 (5.6)
	Asian		9 (6.6)	22 (14.6)	31 (11.0)	9 (8.8)	23 (12.5)	32 (11.0)
	Other		114 (84.4)	106 (71.1)	220 (77.5)	82 (79.6)	127 (69.4)	209 (73.0)
	Unknown		8 (5.9)	13 (8.7)	21 (7.4)	10 (9.7)	19 (10.4)	29 (10.1)
