# Supplementary material for: A novel drug specific mRNA biomarker predictor for selection of patients responding to dovitinib treatment of advanced renal cell carcinoma and other solid tumors
Source: PLoS One. 2023 Aug 30;18(8):e0290681. doi: 10.1371/journal.pone.0290681 (PMC10468037; doi:10.1371/journal.pone.0290681)
Supplement: S3 Table — (PDF) [file pone.0290681.s003.pdf]

S3 Table: Baseline performance status

		Dovitinib			Sorafenib		
		Assayable N=135	Non-Assayable N=149	All N=284	Assayable N=103	Non-Assayable N=183	All N=286
		n (%)					
Karnofsky	100	43 (31.9)	40 (26.8)	83 (29.2)	23 (22.3)	50 (27.3)	73 (25.5)
	90	45 (33.3)	48 (32.2)	93 (32.7)	40 (38.8)	61 (33.3)	103 (35.3)
	80	33 (24.4)	40 (26.8)	73 (25.7)	29 (28.2)	54 (29.5)	83 (29.0)
	70	14 (10.4)	21 (14.1)	35 (12.3)	11 (10.7)	18 (9.8)	29 (10.1)
		n (%)					
MSKCC	Favorable	29 (21.5)	29 (19.5)	58 (20.4)	20 (19.4)	39 (21.3)	59 (20.6)
	Intermediate	79 (58.5)	85 (57.0)	164 (57.7)	59 (57.3)	103 (56.3)	162 (56.6)
	Poor	27 (20.0)	35 (23.5)	62 (21.8)	24 (23.3)	41 (22.4)	65 (22.7)
