# Supplementary material for: A novel drug specific mRNA biomarker predictor for selection of patients responding to dovitinib treatment of advanced renal cell carcinoma and other solid tumors
Source: PLoS One. 2023 Aug 30;18(8):e0290681. doi: 10.1371/journal.pone.0290681 (PMC10468037; doi:10.1371/journal.pone.0290681)
Supplement: S4 Table — (PDF) [file pone.0290681.s004.pdf]

S4 Table: Metastatic sites

	Dovitinib			Sorafenib		
Metastatic Sites	Assayable N=135	Non-Assayable N=149	All N=284	Assayable N=103	Non-Assayable N=183	All N=286
	n (%)					
Bone	40 (29.6)	59 (39.6)	99 (34.9)	50 (48.5)	69 (37.7)	119 (41.6)
Liver	40 (29.6)	54 (36.2)	94 (33.1)	28 (27.2)	66 (36.1)	94 (32.9)
Lung	102 (75.6)	122 (81.9)	224 (78.9)	77 (74.8)	139 (76.0)	216 (75.5)
Lymph Nodes	71 (52.6)	73 (49.0)	144 (50.7)	50 (48.5)	97 (53.0)	147 (51.4)
Other	92 (68.1)	90 (60.4)	182 (64.1)	74 (71.8)	131 (71.6)	205 (71.7)
Missing	2 (1.5)	1 (0.7)	3 (1.1)	2 (1.9)	1 (0.5)	3 (1.0)
