# Supplementary material for: A novel drug specific mRNA biomarker predictor for selection of patients responding to dovitinib treatment of advanced renal cell carcinoma and other solid tumors
Source: PLoS One. 2023 Aug 30;18(8):e0290681. doi: 10.1371/journal.pone.0290681 (PMC10468037; doi:10.1371/journal.pone.0290681)
Supplement: S5 Table — (PDF) [file pone.0290681.s005.pdf]

S5 Table: Balance between MSKCC risk groups in different arms of the study

MSKCC risk group level	Dovitinib DRP Positive N = 49 n (%)	Dovitinib Biopsy Subgroup N= 135 N (%)	Sorafenib Unselected N = 286 n (%)
Favorable	18 (36.7)	29 (21.5)	59 (20.6)
Intermediate	23 (46.9)	79 (58.5)	162 (56.6)
Poor	8 (16.3)	27 (20.0)	65 (22.7)
