# Supplementary material for: A novel drug specific mRNA biomarker predictor for selection of patients responding to dovitinib treatment of advanced renal cell carcinoma and other solid tumors
Source: PLoS One. 2023 Aug 30;18(8):e0290681. doi: 10.1371/journal.pone.0290681 (PMC10468037; doi:10.1371/journal.pone.0290681)
Supplement: S6 Table — (PDF) [file pone.0290681.s006.pdf]

S6 Table: Stratification of median OS by MSKCC risk factor and treatment arm with 95% CI

MSKCC Risk Group Level	DRP Dovitinib Positive Arm	Sorafenib Unselected Arm
**Favorable**	22.9 (14.7-NE)	19.3 months (13.3-23.9)
**Intermediate**	12.2 months (7.9-27.0)	12.5 months (9.7-14.6)
**Poor**	10.3 months (3.7-26.3)	6.6 months (5.3-9.1)
