# Supplementary material for: A novel drug specific mRNA biomarker predictor for selection of patients responding to dovitinib treatment of advanced renal cell carcinoma and other solid tumors
Source: PLoS One. 2023 Aug 30;18(8):e0290681. doi: 10.1371/journal.pone.0290681 (PMC10468037; doi:10.1371/journal.pone.0290681)
Supplement: S7 Table — (PDF) [file pone.0290681.s007.pdf]

S7 Table: Overview of adverse events (safety evaluable patients)

Parameter	DRP Dovitinib Positive N = 47 n (%)	Sorafenib Unselected N = 284 n(%)
Number of Subjects With ≥ 1 TEAE (All grades)	46 (97.9)	278 (97.9)
Number of Subjects With ≥ 1 Grade 3/4 TEAE	36 (76.6)	201 (71.5)
Number of Subjects With ≥ 1 Related TEAE	43 (91.5)	258 (90.8)
Number of Subjects With ≥ 1 SAE	22 (46.8)	130 (45.8)
Number of Subjects With ≥ 1 Related SAE	9 (19.1)	36 (12.7)
Number of Subjects With ≥ 1 TEAE Leading to Study Treatment Discontinuation	12 (25.5)	57 (20.1)
Number of Subjects With ≥ 1 TEAE Leading to Dose Adjustment or Study Treatment Interruption	27 (57.4)	141 (49.6)
Number of Subjects With ≥ 1 TEAE Leading to Death	6 (12.8)	47 (16.5)
